# Annual acknowledgement of manuscript reviewers

**DOI:** 10.1186/1479-5868-10-32

**Published:** 2013-03-19

**Authors:** Frank van Lenthe

**Affiliations:** 1 Department of Public Health, , Erasmus MC, Rotterdam, Netherlands

## Abstract

**Contributing reviewers:**

The editors of International Journal of Behavioral Nutrition and Physical Activity would like to thank all our reviewers who have contributed to the journal in Volume 9 (2012).

## 

Anna Adachi-Mejia

United States of America

Barbara Ainsworth

United States of America

Annie Anderson

United Kingdom

Robert Andrews

United Kingdom

James Annesi

United States of America

Stephen Anton

United States of America

Marni Armstrong

Canada

Lauren Arundell

Australia

Audie Atienza

United States of America

Hannah Badland

Australia

Surinder Baines

Australia

Isabel Balaguer

Spain

Kylie Ball

Australia

Tom Baranowski

United States of America

Jacob Barkley

United States of America

Daheia Barr-Anderson

United States of America

Katherine Bauer

United States of America

Adrian Bauman

Australia

Mathieu Belanger

Canada

Elling Bere

Norway

Christina Berg

Sweden

Jerica Berge

United States of America

Patrick Bergman

Sweden

Sveinung Berntsen

Norway

Kirsten Bevelander

Netherlands

Jennifer Bhalla

United States of America

Stuart Biddle

United Kingdom

Amanda Birnbaum

United States of America

Jackie Blissett

United Kingdom

Beth Bock

United States of America

Filip Boen

Belgium

Christopher Boone

United States of America

Janne Boone-Heinonen

United States of America

Leah Brennan

Australia

Rowan Brockman

United Kingdom

Helen Brown

Australia

Christoph Buck

Germany

Flavia Bürgi

Switzerland

Deborah Burnet

United States of America

Carol Byrd-Bredbenner

United States of America

Robin Callister

Australia

Adrian Cameron

Australia

Jason Cao

United States of America

Cristina Caperchione

Canada

Greet Cardon

Belgium

Jordan Carlson

United States of America

Valerie Carson

Canada

Alison Carver

Australia

Darla Castelli

United States of America

Nick Cavill

United Kingdom

Carlos Celis Morales

United Kingdom

Frank Chaloupka

United States of America

Nikos Chatzisarantis

Singapore

Nigel Chaumeton

United States of America

Palma Chillón

Spain

Mai Jeanette Maidy Chinapaw

Netherlands

Leena Choi

United States of America

Bronwyn Clark

Australia

Verity Cleland

Australia

Natalie Colabianchi

United States of America

Isobel Contento

United States of America

Terry Conway

United States of America

Emma Coombes

United Kingdom

Kristen Copeland

United States of America

Jennifer Copeland

Canada

Kirsten Corder

United Kingdom

Nadia Corsini

Australia

David Crawford

Australia

Noe Crespo

United States of America

Scott Crouter

United States of America

Rik Crutzen

Netherlands

Karen Cullen

United States of America

Steven Cummins

United Kingdom

Philippa Dall

United Kingdom

Catherine Davis

United States of America

Kirsten Davison

United States of America

Ilse De Bourdeaudhuij

Belgium

Katrien De Cocker

Belgium

Stefaan De Henauw

Belgium

Elske de Jong

Netherlands

Marleen de Moor

Netherlands

Nanne de Vries

Netherlands

Barbara Dennison

United States of America

Cassandra Diep

United States of America

Marsha Dowda

United States of America

Clemens Drenowatz

Germany

Scott Duncan

New Zealand

Dustin Duncan

United States of America

David Dunstan

Australia

Genevieve Dunton

United States of America

Elizabeth Eakin

Australia

Robert Eckel

United States of America

Mark Edwards

United Kingdom

Eric Emerson

United Kingdom

Leonard H. Epstein

United States of America

Dale Esliger

United Kingdom

Charlotte Evans

United Kingdom

Kelly Evenson

United States of America

Frank Eves

United Kingdom

Nicole Ezendam

Netherlands

Stuart Fairclough

United Kingdom

Meghan Finch

Australia

Kevin Finn

United States of America

Myron Floyd

United States of America

Ann Forsyth

United States of America

Charlie Foster

United Kingdom

Sarah Foster

Australia

Annika Frahsa

Germany

Patty Freedson

United States of America

Kim Gans

United States of America

Ronnesia Gaskins

United States of America

Klaus Gebel

Australia

Lisa Gibbs

Australia

Janneke Giesen

Netherlands

Gaston Godin

Canada

Rebecca Golley

Australia

Lora Suzanne Goodell

United States of America

Margaret Grant

United Kingdom

Carola Grebitus

Germany

Melanie Green

United States of America

Anders Grøntved

Denmark

Jessica Sophia Gubbels

Netherlands

Joanne Guthrie

United States of America

Leen Haerens

Belgium

Eric Hall

United States of America

Jude Hancock

United Kingdom

Susan Handy

United States of America

Charlotte Hardman

United Kingdom

Lisa Harnack

United States of America

Flo Harrison

United Kingdom

Ellen Haug

Norway

Severin Haug

Switzerland

Marquis Hawkins

United States of America

Emma Haycraft

United Kingdom

Genevieve Healy

Australia

Erik Hemmingsson

Sweden

Marike Reinier Catherine Hendriks

Netherlands

C. Peter Herman

Canada

Kylie Hesketh

Australia

Melanie Hingle

United States of America

Trina Hinkley

Australia

Trina Hinkley

Australia

Mirja Hirvensalo

Finland

Anette Hjartåker

Norway

Eric Hodges

United States of America

Caroline Horwath

New Zealand

Inge Houkes

Netherlands

Ciska Hoving

Netherlands

Ya Jun Huang

Hong Kong

Anne Hublet

Belgium

Sheryl O. Hughes

United States of America

Anita Hurtig-Wennlof

Sweden

Xanne Janssen

Australia

Majken Jensen

United States of America

Karen Jetter

United States of America

Rachel Jones

Australia

Andy Jones

United Kingdom

Carlijn Kamphuis

Netherlands

Ashima Kant

United States of America

Ruth Kipping

United Kingdom

Alison Kirk

United Kingdom

Heather Kitzman-Ulrich

United States of America

Linda Knol

United States of America

Harold Kohl

United States of America

Ferman Konukman

United States of America

Helena Kraemer

United States of America

Magdalena Kwasniewska

Poland

Jouni Lahti

Finland

Junilla Kirsten Larsen

Netherlands

Patrick W. C. Lau

Hong Kong

Rebecca Lawton

United Kingdom

Tracey Ledoux

United States of America

Rebecca Lee

United States of America

Amanda Lee

Australia

Ioni Lewis

Australia

Kaigang Li

United States of America

Djin Gie Liem

Australia

Sonia Lippke

Netherlands

Sam Logan

United States of America

Chris Lonsdale

Australia

Lobo Louie

Hong Kong

Amy Shirong Lu

United States of America

David Lubans

Australia

Julie Lumeng

United States of America

Kyle Macdonald-Wallis

United Kingdom

Duncan Macfarlane

Hong Kong

Ralph Maddison

New Zealand

Bernard Maire

France

Tomi Mäkinen

Finland

David Markland

United Kingdom

Simon Marshall

United States of America

Brian C. Martinson

Afghanistan

Ference Marton

Sweden

Louise C. Masse

Canada

Charles Matthews

United States of America

Mario Mazzocchi

Italy

Noreen McDonald

United States of America

Kelsey McDonald

United States of America

Suzanne McGregor

United Kingdom

M. Diane McKee

United States of America

Rachael McLean

New Zealand

Robert McMurray

United States of America

Sarah McNaughton

Australia

Jessie Meis

Netherlands

Robin Mellecker

Hong Kong

Jason Mendoza

United States of America

Dafna Merom

Australia

Ursina Meyer

Switzerland

Julie Midtgaard

Denmark

Ann Millard

United States of America

Richard Mitchell

United Kingdom

Lorenzo Monasta

Italy

Graham Moore

United Kingdom

Jorge Mota

Portugal

Jean-Claude Moubarac

Brazil

Kerry Mummery

Canada

Dara Musher-Eizenman

United States of America

Ina Nehring

Germany

Gina Netto

United Kingdom

Dianne Neumark-Sztainer

United States of America

Cliona Ni Mhurchu

New Zealand

Virginie Nicaise

United States of America

Theresa Nicklas

United States of America

Robert Noland

United States of America

Kate Northstone

United Kingdom

Nicole Novak

United States of America

Gisela Nyberg

Sweden

Teresia O'Connor

United States of America

Tim Olds

Australia

Christine Olson

United States of America

Jean-Michel Oppert

France

Angeliki Papadaki

United Kingdom

Song-Yi Park

United States of America

Russell Pate

United States of America

Heather Patrick

United States of America

Susan Paxton

Australia

Jamie Pearce

United Kingdom

Geeske Peeters

Australia

Louisa Peralta

Australia

George Ploubidis

United Kingdom

Lisa Powell

United States of America

Thomas Power

United States of America

Stephanie Prince

Canada

Richard G. Prins

Netherlands

Lieke Raaijmakers

Netherlands

Gail Regan

United States of America

Carry Renders

Netherlands

Kyung Rhee

United States of America

Ryan Rhodes

Canada

Kate Ridley

Australia

Deborah Riebe

United States of America

William Riley

United States of America

Chris Rissel

Australia

Christina Roberto

United States of America

Jennifer Robertson-Wilson

Canada

Eric Robinson

United Kingdom

Dori Rosenberg

United States of America

Nancy Ross

Canada

Joseph Rossi

United States of America

Alex Rowlands

United Kingdom

Louise Russell

United States of America

Geert Rutten

Netherlands

Arja Sääkslahti

Finland

Brian Saelens

United States of America

Shannon Sahlqvist

Australia

James Sallis

United States of America

Jo Salmon

Australia

Oddrun Samdal

Norway

Paul Sanderson

United Kingdom

Travis Saunders

Canada

Peter Schantz

Sweden

Patrick Schneider

United States of America

Daniela Schulz

Netherlands

Simon Sebire

United Kingdom

Lauren Sherar

Canada

Brian Shiner

United States of America

Niamh Shortt

United Kingdom

Madeleine Sigman-Grant

United States of America

Helen Skouteris

Australia

Eline Smit

Netherlands

Dianna Smith

United Kingdom

Shawn Somerset

Australia

Kendrin Sonneville

United States of America

John Spence

Canada

Alison Spence

Australia

Seth Spielman

United States of America

Andrew Springer

United States of America

Jamie Stang

United States of America

Rebekah Steele

Australia

Jorien Strijk

Netherlands

Takemi Sugiyama

Australia

Jonas Swartz

United States of America

Deborah Tate

United States of America

Rachael Taylor

New Zealand

Saskia Te Velde

Netherlands

Pedro Teixeira

Portugal

Jennifer Temple

United States of America

Gill Ten Hoor

Netherlands

Megan Teychenne

Australia

Cecilie Thøgersen-Ntoumani

United Kingdom

Debbe Thompson

United States of America

Anne Thorndike

United States of America

Anna Timperio

Australia

Sandar Tin Tin

New Zealand

Alison Tovar

United States of America

Stewart Trost

United States of America

Stewart Trost

Australia

Larry Tucker

United States of America

Gavin Turrell

Australia

Léonie Uijtdewilligen

Netherlands

Patricia van Assema

Netherlands

Rob van Dam

Singapore

Bart van den Borne

Netherlands

Delfien Van Dyck

Belgium

Dave Van Kann

Netherlands

Mireille van Poppel

Netherlands

Maartje van Stralen

Netherlands

Corneel Vandelanotte

Australia

Elizabeth Vandewater

United States of America

Alison Ventura

United States of America

Maite Verloigne

Belgium

Victor Viana

Portugal

Kristin Vickers

Canada

Tommy Visscher

Netherlands

Eline Vlasblom

Netherlands

Susan Walker

United Kingdom

May Wang

United States of America

Dianne Ward

United States of America

Jane Wardle

United Kingdom

Tracy Washington

Australia

Wilma E Waterlander

New Zealand

Kathleen Watson

United States of America

Ailsa Welch

United Kingdom

Greg Welk

United States of America

Josefine Wendel

United States of America

Melicia Whitt-Glover

United States of America

Katrien Wijndaele

United Kingdom

Geoffrey Williams

United States of America

Philip Michael Wilson

Canada

Donna Wilson

Canada

Janet Withall

United Kingdom

Bente Wold

Norway

Catherine Woods

Ireland

John Wright

United States of America

Kiyoshi Yamaki

United States of America

Deborah Young

United States of America

Issa Zakeri

United States of America

Cathleen Zick

United States of America

